# Co‐occurrence of a novel *PDGFRB* variant and likely pathogenic variant in *CASR* in an individual with extensive intracranial calcifications and hypocalcaemia

**DOI:** 10.1002/ccr3.1265

**Published:** 2017-11-20

**Authors:** Natasha N. DeMeo, Jeremy D. Burgess, Patrick R. Blackburn, Jennifer M. Gass, John Richter, Herjot K. Atwal, Jay A. van Gerpen, Paldeep S. Atwal

**Affiliations:** ^1^ Department of Neuroscience Mayo Clinic Jacksonville Florida; ^2^ Center for Individualized Medicine Mayo Clinic Jacksonville Florida; ^3^ Department of Health Sciences Research Mayo Clinic Jacksonville Florida; ^4^ Department of Clinical Genomics Mayo Clinic Jacksonville Florida; ^5^ Department of Pharmacy Mayo Clinic Jacksonville Florida; ^6^ Department of Neurology Mayo Clinic Jacksonville Florida

**Keywords:** *CASR*, Fahr's syndrome, autosomal dominant, hypocalcaemia, *PDGFRB*, primary familial brain calcification, whole‐exome sequencing

## Abstract

This case report describes an individual with brain calcifications, cognitive decline, motor dysfunction, and hypocalcaemia. Exome sequencing revealed a previously reported variant in the *CASR* gene and a variant of uncertain significance in *PDGFRB*. The clinical phenotype is likely explained by the *CASR* variant, but we discuss how the *PDGFRB* variant could also participate in the phenotype.

## Introduction

Calcifications of the basal ganglia were first described in 1850 1, and since that time intracranial calcifications have been linked to multiple disorders and even incidentally discovered at a low frequency within healthy populations 2, 3, 4, 5. Primary familial brain calcification (PFBC, or Fahr's syndrome) is a primarily autosomal dominant disorder characterized by progressive, peri‐capillary bilateral calcifications of the brain, especially in the basal ganglia, but also seen in the cerebellum, thalami, brainstem, and subcortical white matter 3, 6, 7. To identify PFBC, clinicians rely on several core criteria to diagnose patients, including progressive neurological and/or psychological symptoms, the elimination of other underlying etiologies, such as trauma or metabolic disorders, and a family history of basal ganglia calcification 3, 7, 8.

Despite these guidelines, reported phenotypes vary considerably 3, 8, and there is possible confusion with other calcification disorders due to over 35 different descriptive terms being used within the literature 3. With the increased utilization of clinical genetic testing, this diagnostic uncertainty has been reduced, and several genes have been linked to PFBC in recent years 9, 10, 11, 12. Here, we report a novel variant of uncertain significance (VUS) in the platelet‐derived growth factor receptor beta subunit (*PDGFRB*) gene as well as a likely pathogenic variant of the calcium‐sensing receptor (*CASR*) gene in the same individual.

## Materials and Methods

### Ethical compliance

The patient consented to sample collection and subsequent analysis under a protocol approved by the institutional review board of the Mayo Clinic. Written informed consent was obtained from the patient for publication and accompanying images.

### Whole‐exome sequencing

Clinical whole‐exome sequencing (WES) was performed by GeneDx (XomeDxPlus). Briefly, genomic DNA was extracted from the proband. As described in the clinical testing methodology by GeneDx, the Agilent Clinical Research Exome capture kit was used for exome enrichment and sequencing was performed on an Illumina HiSeq 2000 that generates 100 bp paired‐end reads. Bidirectional sequences were assembled, aligned to reference gene sequences based on human genome build GRCh37/UCSC hg19, and analyzed for sequence variants using a proprietary analysis tool (Xome Analyzer, GeneDx). Sanger sequencing was used to confirm all disease relevant variants identified in this individual. Variant filtration techniques were performed per standard commercial methods for GeneDx. Sequence alterations were reported according to the Human Genome Variation Society (HGVS) nomenclature guidelines.

## Clinical Report

### Case description

The patient is a 73‐year‐old man of northern European origin, who presented with a several year history of progressive dysarthria and mild aphasia. The patient reported that he was “…not able to put sentences together.” He further asserted that he had “lost [his] voice,” following each of two previous hip replacement surgeries. Similarly, laryngospasm and tetany were reported. The patient's neurological examination revealed mild to moderate cognitive impairment, characterized by deficits in concentration, executive dysfunction, diminished visual constructional and visuospatial skills, slowed verbal fluency, diminished naming, and inefficient memory retention. These findings were consistent with mild cognitive impairment, secondary to frontal, temporal, and parietal lobe dysfunction. Speech and language assessments revealed a mixed dysarthria with hyperkinetic and hypokinetic features. There was also mild oral apraxia and mild impairment in motor programming.

**Figure 1:**
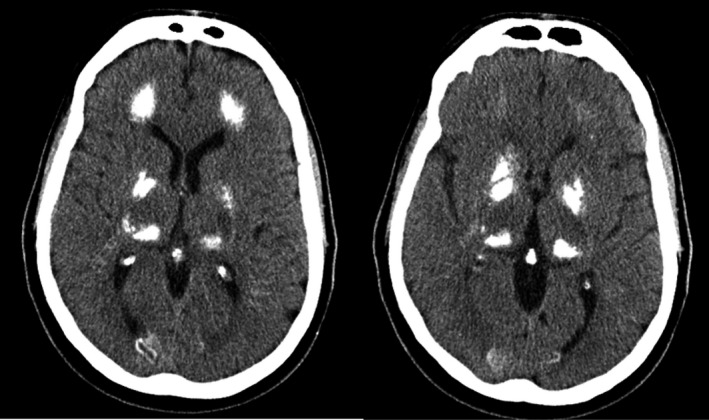
CT scan in the axial plane showing extensive bilateral calcification of basal ganglia.

His most recent imaging examinations (Fig. 1) revealed symmetric, bilateral calcifications of the cerebral deep white matter, basal ganglia, thalami, medial occipital cortices, and deep cerebellar white matter. There was no evidence of atrophy. Prior computed tomography (CT) and magnetic resonance imaging (MRI) examinations also detected extensive calcifications, notably of the basal ganglia, but also across other regions. On MRI, this manifested as increased signal in T1‐weighted images in the corona radiata, centrum semiovale, thalami, dentate nuclei, and cerebellar white matter. In these earlier images, there was evidence of atrophy reported that disproportionately affected the frontal and parietal lobes, and to a milder extent, the cerebellum. The patient had previously undergone MRI in 2009 after suffering a stroke. His records from this time make no mention of brain calcifications, suggesting that this finding was progressive and has persisted for at least 2 years.

Given the link between basal ganglia calcification and metabolic/endocrine dysregulation, the patient was tested for hypoparathyroidism. He displayed low calcium at 8.6 mg/dL (reference range ≥22 years: 8.9–10.1 mg/dL), but creatinine, parathyroid hormone (PTH), and thyroid‐stimulating hormone (TSH) were within normal ranges.

### Genetic testing

**Figure 2:**
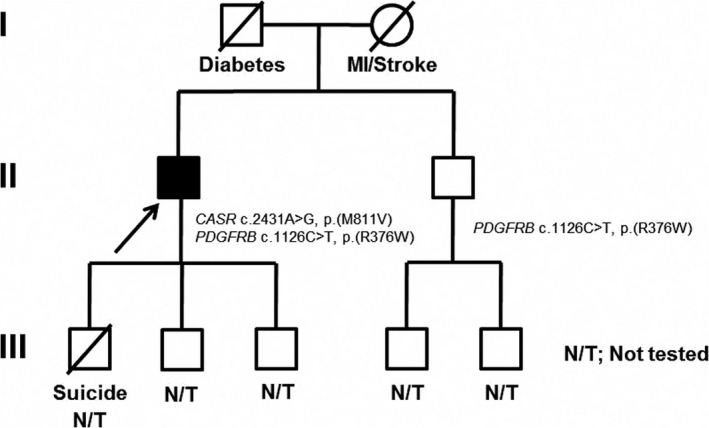
Three‐generation family pedigree. Proband indicated with arrow. Both the proband and his asymptomatic brother share a PDGFRB variant, whilst the proband additionally carries the CASR variant.

There were no related clinical findings in the proband's family (see Fig. 2), although his paternal grandfather and son are reported to have either attempted (former) or committed suicide (latter). Notably, depression has been linked with PFBC. The patient also has a son with a history of Graves’ disease with hyperthyroidism. Previous testing by Fulgent for two of the most common PFBC‐associated genes (*PDGFB* and *SLC20A2*) did not detect any variants, therefore the patient underwent WES through the Mayo Clinic Department of Clinical Genomics. WES revealed variants in two genes (Table 1) possibly associated with the patient's phenotype: a likely pathogenic variant in the *CASR* gene (Chr3(GRCh37): g.122003232A>G, NM_000388.2: c.2431A>G, NP_000379.2: p.(Met811Val)) and a novel variant of uncertain significance in *PDGFRB* (Chr5(GRCh37): g.149512314G>A, NM_002609.3: c.1126C>T, NP_002600.1: p.(Arg376Trp)). The p.(M811V) variant in exon 7 of *CASR* was not observed in approximately 123,136 exomes and 15,496 genomes in the Genome Aggregation Database (gnomAD). The variant results in a conservative amino acid substitution, which occurs at a position that is conserved across species, down to *Danio rerio* (zebrafish). *In silico* analysis predicts this variant is likely damaging to the protein structure and/or function (see Table 2). The p.(R376W) variant in *PDGFRB* was seen in 3/272342 alleles in gnomAD 13; http://gnomad.broadinstitute.org/). The variant results in a nonconservative amino acid substitution, which is predicted to impact secondary protein structure. The variant falls at the end of exon 7, but is not predicted to impact splicing based on *in silico* splice prediction tools. This substitution occurs at a position that is moderately conserved across species (including in *Gallus gallus*). *In silico* analysis 14 predicts this variant is likely damaging to the protein structure and/or function (see Table 2).

**Table 1:** Potentially pathogenic variants identified in genes related to the patient's condition through WES

Gene	Diseases (MIM #)	Mode of Inheritance	Variant	Coding DNA	Zygosity	Mode of Inheritance	gnomAD frequency	dbSNP	ClinVar Accession number	Classification
CASR	239200; 601198; 145980; 612899	Autosomal Dominant/Recessive	p.(M811V)	c.2431A>G	Heterozygous	Unknown	Not reported	Not reported	SCV000521154	Likely Pathogenic Variant
PDGFRB	615007; 616592; 131440; 228550; 601812	Autosomal Dominant	p.(R376W)	c.1126C>T	Heterozygous	Unknown	3/272342 (0.00001102%)	rs142621427	None	Variant of Uncertain Significance

**Table 2:** Predicted SNP effects for the identified variants from *in silico* analysis using PredictSNP2 14

	Tool	PredictSNP2	CADD	DANN	FATHMM	FunSeq2	GWAVA
CASR chr3:122003232 A>G	Prediction	Deleterious	Deleterious	Deleterious	Deleterious	Neutral	Unknown
	Score	1	21.4	0.9954	0.9901	2	0.48
	Exp. accuracy	0.87	0.54	0.6	0.83	0.62	0.48
PDGFRB chr5:149512314 G>A	Prediction	Deleterious	Deleterious	Deleterious	Deleterious	Deleterious	Deleterious
	Score	1	23.1	0.9982	0.9021	3	0.26
	Exp. accuracy	0.87	0.55	0.7	0.56	0.61	0.51

The proband's asymptomatic 72‐year‐old brother was also tested and found to have the *PDGFRB* variant but not the *CASR* (see pedigree in Fig. 2) variant.

## Discussion

Intracranial mineral deposition is common to a number of disorders, including certain neurodegenerative and metabolic diseases, although it also occurs at low levels in the general population and may be related to the normal aging process 2, 3, 4, 5. In PFBC, calcium is deposited in perivascular areas of the brain, especially the basal ganglia and cerebellar white matter, and is most commonly linked to movement and cognitive dysfunctions 3, 6, 8, 15.

In this case study, multiple MRI and head CT scans uncovered extensive bilateral calcifications in a patient originally presenting with dysarthria and reductions in learning and memory abilities. Consistent with PFBC, the patient's calcifications were found in the deep white matter of the cerebrum and cerebellum, thalami, and basal ganglia. WES uncovered two heterozygous genetic variants in this patient: one novel VUS in *PDGFRB* and one likely pathogenic variant in *CASR*.

The first gene, *PDGFRB* (MIM #173410), produces a homodimeric tyrosine kinase (TK) receptor (PDGF‐R*β*) that can bind multiple ligands, including PDGF‐B (platelet‐derived growth factor subunit B), which, along with the receptor, has been linked to PFBC in a number of recent studies 6, 9, 11, 16, 17, 18, 19. PDGF‐R*β* is thought to be involved in angiogenesis and hematopoiesis during development, and Keller et al. 9 proposed that the accumulation of calcium in PDGF‐R*β*/PDGF‐B linked PFBC cases may be related to a dysfunctional blood–brain barrier (BBB).

Most reported cases of *PDGFRB* variants have been classified as gain‐of‐function, leading to disorders such as infantile myofibromatosis (MIM #228550), Kosaki overgrowth disorder (MIM #616592) 20, and premature aging disorders (MIM #601812) 21. However, several variants in or near the split‐TK domain of the receptor have been found in patients with basal ganglia calcifications 6, 11, 18, 19. Physically closest to our novel variant (c.1126C>T, p.(R376W)) is a missense variant at codon 371 (p.N371K, c.1113C>G), although the patient in this case was diagnosed with Cornelia de Lange syndrome and had no mention of brain calcifications 22. Lastly, whilst our variant falls close to a splice site, splicing predictions were not in favor of a strong effect on splicing.

The second genetic variant uncovered by WES in the *CASR* gene (c.2431A>G, p.(M811V)) (MIM #601199) has been reported previously in a family with hypoparathyroidism, hypocalcaemia, and relative hypercalciuria 23. Similarly, the proband described here has hypocalcaemia and borderline low‐normal parathyroid hormone and serum magnesium levels, which is characteristic of autosomal dominant hypocalcaemia caused by an activating *CASR* variant 24. The variant was shown to segregate with the hypocalcaemic phenotype in three generations of affected individuals in this family, while unaffected family members were negative for the variant, providing strong evidence for its pathogenicity. Activating *CASR* variants lead to defective sensing and regulation of serum calcium levels, resulting in a reduction in parathyroid hormone and maintained hypocalcaemia. Interestingly, 50% of patients presenting with hypercalciuric hypocalcaemia syndrome type 1 are estimated to have asymptomatic hypocalcaemia, and over 35% have ectopic and/or basal ganglia calcifications 24.

As PFBC is partially diagnosed based on the exclusion of underlying metabolic etiology 3, 7, 8, the presence of hypocalcaemia combined with two potentially contributing genetic variants makes diagnosis difficult. It is possible that both variants contribute to this patient's complex phenotype. For example, in one reported case, a patient rapidly developed extensive unilateral calcifications in both the white and gray matter several weeks after an ischemic stroke in the same brain region 25. The rapid, ectopic accumulation of calcium was thought to occur due to the pre‐existing imbalanced ratio of phosphate to calcium and the weakened vasculature at the site of the stroke 25. This report by Wityk, combined with findings of disrupted BBBs in both mice with *PDGF‐B* variants 9 and postmortem PFBC human tissue 26, strengthens the hypothesis that an impaired BBB/vascular system could lead to increased susceptibility to mineral deposition in the brain, especially when combined with metabolic imbalances. However, metabolic dysfunction has been reported to cause ectopic mineral deposition independently of any reported increase in BBB or vascular vulnerability, as seen by Kurozumi et al. 27 and Schouten et al. 23. Additionally, a mouse model experiment performed by Vanlandewijck et al. 28 found that PGDFB^ret/ret^ mice actually had healthier BBBs in brain regions prone to calcification as compared to regions not prone to calcification 28. The association between BBB impairment and increased mineralization must then merit further investigation.

In this report, the clinical presentation of the patient seems to more closely fit that of PFBC compared to that of activating *CASR* variants, although his hypocalcaemia is certainly attributable to the latter. In PFBC, the most commonly reported manifestation is movement disorder, closely followed by cognitive and cerebellar impairments, and speech disorder 3, 6, 8, 9. In this case, the patient originally sought medical attention due to cognitive decline and speech impairment, although some tremulous movements in his upper limbs, muscular fasciculations, and dysphagia were noted during examination. Other less commonly reported symptoms of PFBC include stroke 6, 15 and vertigo 29, both of which were identified in this patient prior to or during treatment. In this case, the vertigo was successfully ameliorated using Epley chair repositioning. Additionally, Mufaddel & Al‐Hassani 8 reported that in 40% of patients, psychiatric features are often the first presenting symptom of PFBC and the most common of these is depression. Interestingly, although the patient asserts that he has no current or previous episodes of depression, there is a documented family history of this condition and the patient notes that he has had increased emotional reactivity in recent years.

Follow‐up genetic testing of the patient's asymptomatic 72‐year‐old brother revealed the same heterozygous variant in *PDGFRB* (c.1126C>T, p.(R376W)), but no changes in *CASR* were detected. However, this information is the extent of available family history, as no other testing, including CT imaging, was completed for the brother, and other family members were unavailable for testing. Consequentially, we were unable to use the brother's information to determine if the isolated *PDGFRB* variant is associated with any specific features of the patient's phenotype. The lack of familial confirmation is the largest limitation of the current report as it is impossible to determine if PFBC or hypocalcaemia are present in untested members of the family, the mode of inheritance, and whether either of the variants segregate with the ectopic calcifications, cognitive symptoms, and/or hypocalcaemia as reported in the proband. The limited information provided by the brother's genetic testing and his apparent lack of symptoms may provide clues as to the causative variant in the proband; however, incomplete penetrance and a variable age of onset have commonly been reported for PFBC 3, 6, 15, 30. Of note, PFBC and hypocalcaemia caused by *CASR* changes are thought to be inherited mainly via an autosomal dominant pattern 9, 19, 23, 26, 27.

## Conclusions

In conclusion, we report a 73‐year‐old man with bilateral basal ganglia calcifications and two variants in *CASR* and *PDGFRB* with a possible blended phenotype. We were unable to gather substantial information on familial phenotypes to further investigate the effects of the *PDGFRB* VUS. Despite this, the existence of past cases of *PDGFRB* variants causing basal ganglia calcifications suggests that the VUS could be implicated in the mineralization present in our proband.

## Authorship

NND and JDB: wrote the manuscript. PRB, JMG, JR and HKA: involved in scientific input and revision. JAvG: collected the clinical data and was involved in scientific input and revision. PSA: is the senior author and was involved in scientific input and revision of the final manuscript.

## Conflict of Interests

The authors have no conflict of interests or disclosures.
